# The Impact of Proband Indication for Genetic Testing on the Uptake of Cascade Testing Among Relatives

**DOI:** 10.3389/fgene.2022.867226

**Published:** 2022-06-16

**Authors:** Tara J. Schmidlen, Sara L. Bristow, Kathryn E. Hatchell, Edward D. Esplin, Robert L. Nussbaum, Eden V. Haverfield

**Affiliations:** Invitae, San Francisco, CA, United States

**Keywords:** cascade testing, genetic testing, diagnostic testing, proactive screening, hereditary cancer syndromes, familial hypercholesterolemia, CDC tier 1 conditions

## Abstract

Although multiple factors can influence the uptake of cascade genetic testing, the impact of proband indication has not been studied. We performed a retrospective, cross-sectional study comparing cascade genetic testing rates among relatives of probands who received either diagnostic germline testing or non-indication-based proactive screening via next-generation sequencing (NGS)-based multigene panels for hereditary cancer syndromes (HCS) and/or familial hypercholesterolemia (FH). The proportion of probands with a medically actionable (positive) finding were calculated based on genes associated with Centers for Disease Control and Prevention (CDC) Tier 1 conditions, HCS genes, and FH genes. Among probands with a positive finding, cascade testing rates and influencing factors were assessed. A total of 270,715 probands were eligible for inclusion in the study (diagnostic *n* = 254,281,93.9%; proactive *n* = 16,434, 6.1%). A positive result in a gene associated with a CDC Tier 1 condition was identified in 10,520 diagnostic probands (4.1%) and 337 proactive probands (2.1%), leading to cascade testing among families of 3,305 diagnostic probands (31.4%) and 36 proactive probands (10.7%) (*p* < 0.0001). A positive result in an HCS gene was returned to 23,272 diagnostic probands (9.4%) and 970 proactive probands (6.1%), leading to cascade testing among families of 6,611 diagnostic probands (28.4%) and 89 proactive probands (9.2%) (*p* < 0.0001). Cascade testing due to a positive result in an HCS gene was more commonly pursued when the diagnostic proband was White, had a finding in a gene associated with a CDC Tier 1 condition, or had a personal history of cancer, or when the proactive proband was female. A positive result in an FH gene was returned to 1,647 diagnostic probands (25.3%) and 67 proactive probands (0.62%), leading to cascade testing among families of 360 diagnostic probands (21.9%) and 4 proactive probands (6.0%) (*p* < 0.01). Consistently higher rates of cascade testing among families of diagnostic probands may be due to a perceived urgency because of personal or family history of disease. Due to the proven clinical benefit of cascade testing, further research on obstacles to systematic implementation and uptake of testing for relatives of any proband with a medically actionable variant is warranted.

## Introduction

Cascade testing is the process of providing genetic counseling and testing to at-risk blood relatives following the detection of a pathogenic variant in a disease-causing gene in a family member (i.e., the proband). Confirming the presence (or absence) of a pathogenic variant in at-risk relatives can inform clinical management, including both preventative measures for unaffected relatives and potential changes in treatment for affected relatives. For example, given a proband with diagnosed breast cancer and a pathogenic variant in *BRCA1,* an unaffected relative who is confirmed to have the same genetic variant may increase mammography screenings or opt for risk-reducing surgery (Daly et al., 2021). The same unaffected relative, if confirmed negative for the pathogenic *BRCA1* variant, could likely forgo escalated screenings or other preventive interventions. A recent study estimated that it would take 9.9 years to detect all carriers of a pathogenic variant in one of 18 genes associated with a hereditary cancer syndrome (HCS) in the United States (including *BRCA1*) if cascade testing were used, compared with 59.5 years if it were not (Offit et al., 2020). Further, cascade testing has the ability to inform reproductive health decisions, especially in relatives who have been identified as carriers of an autosomal recessive disease, and has been demonstrated to be a cost-effective approach for identifying at-risk individuals across many disease types, especially in young, unaffected relatives (Marks et al., 2002; Wonderling et al., 2004; Ademi et al., 2014; Grosse, 2015; Kerr et al., 2017; O’Brien et al., 2021). As a result, cascade testing has immense potential for improving the efficiency of healthcare resource utilization by reducing the burden of care for individuals and families as well as health systems.

Most studies of cascade testing have focused on the genes associated with Tier 1 conditions as established by the Centers for Disease Control and Prevention (CDC), which include hereditary breast and ovarian cancer (HBOC), Lynch syndrome, and familial hypercholesterolemia (FH) (Centers for Disease Control and Prevention, 2021). Evidence demonstrating the utility of cascade testing has led to recommendations and guidelines from professional societies and from the CDC that encourage extending testing to at-risk relatives (Nordestgaard et al., 2013; Hampel et al., 2015; Randall et al., 2017; Committee on Gynecologic Practice, 2018; Sturm et al., 2018; Daly et al., 2021).

Despite mounting evidence on the utility of cascade testing, uptake rates among at-risk relatives remain low overall, though vary across clinical settings (Cernat et al., 2021). Most studies focusing on genes associated with HCS report cascade testing uptake rates between 30 and 60% (Fehniger et al., 2013; Menko et al., 2019; Lee et al., 2021). Uptake rates have been much lower (4–12%) among families with FH in the United States (Ahmad et al., 2016; Gidding et al., 2020; Ajufo et al., 2021), but much higher (30% up to 90%) among families with FH in other Western countries (Marks et al., 2006; Ahmad et al., 2016; van den Heuvel et al., 2020). Limited data are available on cascade testing uptake for proactive or non-indication-based genetic screening, though results from the Electronic Medical Records and Genomics (eMERGE) phase III study demonstrated that only about one-third of probands who received non-indication-based screening reported sharing their test results with their relatives (Wynn et al., 2021). In the present study, we assessed differences in uptake of cascade testing between relatives of probands who received indication-based diagnostic genetic testing and relatives of probands who received proactive, non-indication-based screening for genes associated with HCS or FH.

## Materials and Methods

### Study Population and Design

Two retrospective cohorts of unrelated probands unselected for sex, self-reported ancestry, or age were compiled with individuals who underwent diagnostic germline genetic testing or proactive screening at Invitae from January 2017 through March 2021.

The diagnostic proband cohort included individuals who had clinician-ordered, indication-based testing via the Invitae Common Hereditary Cancers Panel (up to 47 genes) or the Invitae Familial Hypercholesterolemia Panel (up to 4 genes). Specific clinical criteria that led to clinician-ordered testing (e.g., the individual met guidelines from professional societies for testing) were unknown and thus individuals were unselected for test indication (i.e., personally affected versus family history).

The proactive proband cohort included individuals who were referred by clinicians for screening via the Invitae Cancer Screen (up to 61 genes), the Invitae Cardio Screen (up to 77 genes), or the Invitae Genetic Health Screen (up to 147 genes). Genes for inclusion in these panels were selected based on published guidance from the American College of Medical Genetics and Genomics (ACMG) and ClinGen Working groups, in addition to clinical studies establishing personal risk for monogenic disorders (Foreman et al., 2013; Green et al., 2013; Dewey et al., 2016; Webber et al., 2018). Probands undergoing non-indication-based screening have been described previously (Haverfield et al., 2021). In brief, all probands were included in the analysis, regardless of a personal or family history of cancer or cardiovascular disease. Individuals were excluded only if a familial variant associated with a condition on the screening panel had been previously identified.

In both cohorts, if a proband harbored at least one clinically significant variant (including carrier status), then the proband’s relatives were eligible for cascade testing for the identified variant(s). A clinically significant variant was defined as a pathogenic/likely pathogenic (P/LP) variant, a pathogenic-low penetrance (P[LP]) variant, or an increased risk allele (IRA). P(LP) variants are less penetrant compared to other P/LP variants in the same gene and may result in a less obvious Mendelian pattern of inheritance (e.g., *HFE* p.Cys282Tyr or p.His63Asp). IRAs are variants in genes that increase the risk for a condition and have stringent criteria (Ioannidis et al., 2008), but are not associated with a Mendelian inheritance pattern (e.g., *APC* p.Ile1307Lys). Testing was offered at no charge to the relatives for up to 90 days following the proband’s test report date, though the cascade testing window was extended to 150 days after March 30, 2020, due to the COVID-19 pandemic. All blood relatives were eligible for cascade testing, and those who received testing from January 2017 through August 2021 were included in the analysis as long as they were tested for at least one gene in which the proband had a clinically significant variant. Relatives who were tested for the purposes of reclassifying variants of uncertain significance (VUS) in probands within the diagnostic cohort were excluded from the analysis.

Review and analysis of de-identified and aggregated data were approved for waiver of authorization by the WCG Institutional Review Board (study number 1167406).

### Genetic Testing

Requested genes were sequenced via a short-read next-generation sequencing (NGS) assay that used genomic DNA extracted from blood or saliva samples as reported previously (Lincoln et al., 2015; Haverfield et al., 2021). A bioinformatics pipeline aligned sequencing reads and utilized community standard and custom algorithms to identify single nucleotide variants (SNVs), small and large insertions or deletions (indels), structural variants, and exon-level copy-number variants (CNVs) (Lincoln et al., 2015, 2021; Truty et al., 2019).

Detected variants were analyzed and interpreted using Sherloc (Nykamp et al., 2017), a points-based framework that incorporates the joint consensus guidelines from the American College of Medical Genetics and Genomics and the Association for Molecular Pathology (Richards et al., 2015). Based on the evidence, variants were classified as benign or likely benign (B/LB), VUS, P/LP, IRA, or P(LP). Clinically significant P/LP, IRA, and P(LP) variants that did not meet stringent NGS quality metrics were confirmed by an orthogonal assay prior to reporting (Lincoln et al., 2019). For individuals who underwent diagnostic testing, variants classified as P/LP, IRA, P(LP), and VUS were reported. For individuals who underwent proactive screening, only P/LP, IRA, and P(LP) findings were reported, as VUS are not reported as part of proactive screening (Haverfield et al., 2021). All results were returned to the ordering healthcare provider, who then oversaw results disclosure to the individual who underwent diagnostic testing or proactive screening.

Individuals were considered to have “positive” findings with medically actionable results if one clinically significant variant was found in a gene associated with an autosomal dominant disorder or two clinically significant variants were found in a gene associated with an autosomal recessive disorder. In addition, male individuals with one clinically significant variant in any gene associated with an X-linked disorder were considered to have positive findings. Female individuals with one clinically significant variant in a gene associated with an X-linked dominant disorder or two clinically significant variants in a gene associated with an X-linked recessive disorder were considered to have positive findings. A carrier finding was classified as one clinically significant variant in a gene associated with an autosomal recessive disorder in any individual or one clinically significant variant in a gene associated with an X-linked recessive disorder in female individuals. Though all results were disclosed to the ordering clinician first, individuals, regardless of result (e.g., no clinically significant result, medically actionable result), could seek post-test genetic counseling through Invitae, though this was not required.

### Analysis

#### Medically Actionable (Positive) and Clinically Significant (Carrier) Findings in Probands

The proportion of probands with positive and carrier findings were calculated for the diagnostic and proactive cohorts. The three primary comparisons were based on genes that were analyzed in both cohorts (i.e., CDC Tier 1 conditions, HCS, and FH genes). Demographics of each of these groups were also summarized.

Eleven genes associated with CDC Tier 1 conditions (*APOB*, *BRCA1*, *BRCA2*, *EPCAM*, *LDLR*, *LDLRAP1*, *MLH1*, *MSH2*, *MSH6*, *PCSK9*, and *PMS2*) were analyzed in all probands (regardless of panel type). Forty-five HCS genes available to both cohorts were analyzed among patients who underwent diagnostic testing or proactive screening for HCS genes (Supplementary Table S1). Similarly, four FH genes available to both cohorts were analyzed among individuals who underwent testing or screening for FH. Proactive probands who received screening through the Invitae Genetic Health Screen were included in both the HCS and FH cohorts, as this panel included genes across both clinical areas. Diagnostic probands who had both the Invitae Common Hereditary Cancers Panel and the Invitae Familial Hypercholesterolemia Panel ordered were also included in both cohorts.

Additional genes were also analyzed if ordered for probands in either cohort. Diagnostic probands who had the Invitae Common Hereditary Cancers Panel had *CTNNA1* and *RAD50* analyzed. Proactive probands who had the Invitae Cardio Screen or the Genetic Health Screen had up to an additional 72 genes associated with other cardiology-related conditions or up to 16 genes associated with other HCS analyzed. The Invitae Genetic Health Screen also included 10 genes associated with other hereditary diseases (e.g., hereditary hemochromatosis [*HAMP*, *HFE*, *HJV*, *SLC40A1 and TFR2*] and malignant hyperthermia susceptibility [*CACNA1S and RYR1*]) that were analyzed in proactive probands only.

#### Cascade Testing

Among probands with a medically actionable or clinically significant finding, the proportion who had at least one relative undergo cascade testing through Invitae was calculated (i.e., cascade testing rate). Cascade testing uptake rates were compared between the diagnostic and proactive cohorts by calculating the difference in proportion for two independent samples. In addition, the number of relatives tested per proband was analyzed.

Among relatives, demographic characteristics were calculated and stratified according to the proband’s result type (e.g., medically actionable result in a shared HCS gene). Concordance of findings between the relative and proband was assessed.

#### Demographic and Clinical Factors Associated With Cascade Testing Utilization

We also assessed whether any demographic or clinical characteristics of probands influenced the rate of cascade testing among relatives. In both diagnostic and proactive cohorts, probands with medically actionable findings and with relatives who had undergone cascade testing were compared with probands who had a medically actionable finding but did not have relatives who had undergone cascade testing. These two groups were compared based on the following factors: age at time of testing, sex, self-reported ethincity, and whether the proband had a post-test genetic counseling session provided through Invitae. Two additional comparisons were made for probands who underwent diagnostic testing or proactive screening for HCS: whether the gene was associated with a CDC Tier 1 condition (diagnostic and proactive cohorts) and reported personal history of cancer (diagnostic cohort only). Differences in categorical data were assessed by comparing proportions for two independent samples; differences in age were assessed using 2-sample, 2-tailed t-tests. No comparisons were made for the proactive FH cohort due to small sample sizes.

## Results

### Proband Characteristics

A total of 270,715 probands were eligible for inclusion in the study: 254,281 (93.9%) who received indication-based diagnostic testing and 16,434 (6.1%) who received non-indication-based proactive screening (Supplementary Table S2). Diagnostic testing or proactive screening for HCS genes was completed for 247,875 diagnostic probands and 15,984 proactive probands. Diagnostic testing or proactive screening for FH was completed for 6,503 diagnostic probands and 10,776 proactive probands. Of note, 97 diagnostic probands (0.04%) and 10,326 proactive probands (62.8%) had both HCS and FH genes analyzed.

Demographic information for both cohorts based on clinical area is reported in Table 1. Diagnostic probands undergoing genetic testing for HCS were mostly female (87.5%) with a mean age of 55.5 ± 14.5 years. Proactive probands with HCS genes included in the genetic screen were also mostly female (58.0%), with a mean age of 48.4 ± 13.2 years. In both diagnostic and proactive cohorts undergoing testing or screening for FH, approximately half of the probands were female (56.5 and 49.1%, respectively), and the mean ages were 45.0 ± 20.4 years and 48.1 ± 13.0 years, respectively.

**TABLE 1:** Demographic information of probands by clinical area^a^.

	HCS		FH	
	Diagnostic probands (N = 247,875)	Proactive probands (N = 15,984)	Diagnostic probands (N = 6,503)	Proactive probands (N = 10,776)
Sex, n (%)^b^				
Female	216,965 (87.5)	9,265 (58.0)	3,676 (56.5)	5,296 (49.1)
Male	30,908 (12.5)	6,719 (42.0)	2,827 (43.5)	5,480 (50.9)
Age, years				
Mean (SD)	55.5 (14.5)	48.4 (13.2)	45.0 (20.4)	48.1 (13.0)
Median (Q1, Q3)	55 (45, 67)	48 (37,57)	48 (31, 60)	48 (37, 57)
Self-reported ancestry, n (%)				
Ashkenazi Jewish	7,638 (3.1)	606 (3.8)	86 (1.3)	359 (3.3)
Asian	8,009 (3.2)	1,057 (6.6)	318 (4.9)	739 (6.9)
Black	16,829 (6.8)	233 (1.5)	403 (6.2)	135 (1.3)
French-Canadian	313 (0.1)	32 (0.2)	21 (0.3)	28 (0.3)
Hispanic	17,485 (7.1)	441 (2.8)	480 (7.4)	198 (1.8)
Mediterranean	664 (0.3)	148 (0.9)	43 (0.7)	115 (1.1)
Native American	534 (0.2)	10 (0.1)	15 (0.2)	5 (0.05)
Pacific Islander	350 (0.1)	15 (0.1)	16 (0.3)	7 (0.1)
Sephardic Jewish	271 (0.1)	109 (0.7)	5 (0.1)	23 (0.2)
White	160,173 (64.6)	9,700 (60.7)	3,988 (61.3)	6,696 (62.1)
Multiple ancestries	20,668 (8.3)	1,527 (9.6)	432 (6.6)	1,087 (10.1)
Other	3,889 (1.6)	716 (4.5)	162 (2.5)	373 (3.5)
Unknown	11,052 (4.5)	1,390 (8.7)	534 (8.2)	1,011 (9.4)

FH, familial hypercholesterolemia; HCS, hereditary cancer syndrome; Q, quartile; SD, standard deviation.

a Diagnostic probands who had both the Invitae Common Hereditary Cancers Panel and the Invitae Familial Hypercholesterolemia Panel ordered were included in both clinical areas. Proactive probands who had the Invitae Genetic Health Screen were included in the analysis of HCS, and FH, screening results.

b Sex was unknown for two diagnostic probands undergoing HCS testing.

### Positive Findings and Cascade Testing Rates in Genes Associated With CDC Tier 1 Conditions

**FIGURE 1:**
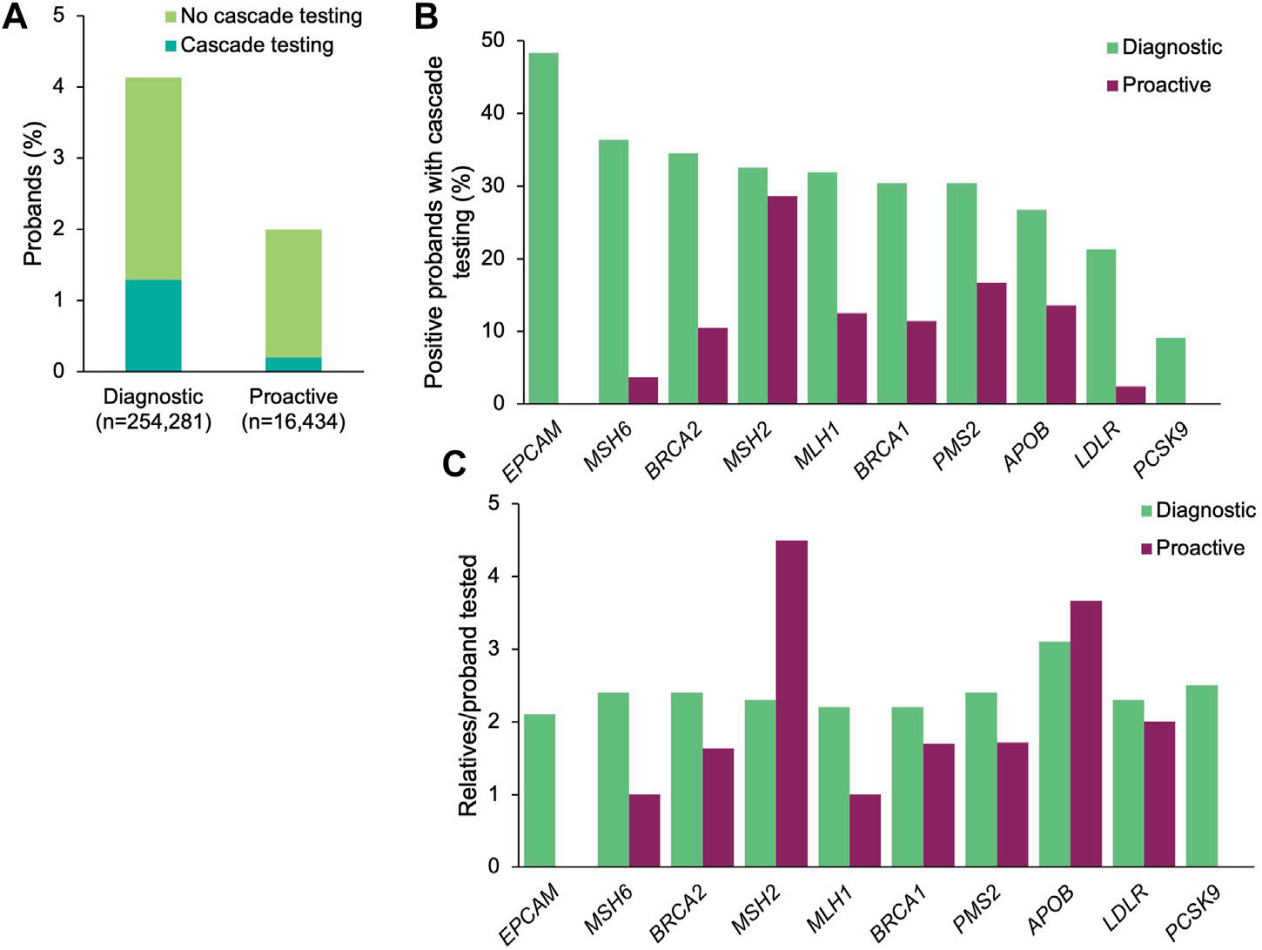
Yield of medically actionable findings in probands among the 11 genes associated with a CDC Tier 1 condition and rates of cascade testing. **(A)** Proportion of diagnostic and proactive probands with a positive result in a gene associated with a CDC Tier 1 condition, stratified by whether cascade testing was pursued. The denominator was the total number of probands that underwent diagnostic testing (*n* = 254,281) or proactive screening (*n* = 16,434). **(B)** Proportion of probands with a medically actionable result in a gene associated with a CDC Tier 1 condition with at least one relative who pursued cascade testing. The denominator was the number of probands with a medically actionable result in each gene associated with a CDC Tier 1 condition for each cohort. Probands with a positive finding in more than one gene associated with a CDC Tier 1 condition were included in calculations for each gene. **(C)** Mean number of relatives who pursued cascade testing per proband with a positive result in a gene associated with a CDC Tier 1 condition. If cascade testing was pursued for positive findings in more than one gene detected in the proband, the relatives and probands were included in the calculations for each gene. CDC, Centers for Disease Control and Prevention.

A positive result in a gene associated with a CDC Tier 1 condition was identified in 10,520 (4.1%) and 337 (2.1%) of the diagnostic and proactive probands, respectively (Figure 1A). The proportion of patients with positive findings varied by gene (Figure 1B). Significantly more diagnostic probands than proactive probands with a positive finding in a gene associated with a CDC Tier 1 condition had at least one relative pursue cascade testing (diagnostic *n* = 3,305, 31.4%; proactive *n* = 36, 10.7%; *p* = 4.76×10^–16^; Figure 1A). Compared to proactive probands, a higher proportion of diagnostic probands with a medically actionable finding in each gene had at least one relative pursue cascade testing, ranging from 9.1 to 48.3% (*vs*. 2.4–28.6% among proactive probands (Figure 1B).

A total of 7,750 relatives of diagnostic probands (2.3 relatives/proband) and 71 relatives of proactive probands (2.0 relatives/proband) underwent cascade testing. The majority of relatives in both cohorts were first-degree relatives (diagnostic 76.1%, *n* = 5,896; proactive 73.2%, *n* = 52), with the remaining being second-degree (10.8%, *n* = 838; 12.7%, *n* = 9), third-degree (5.5%, *n* = 423; 12.7%, *n* = 9), and more distant relatives (7.7%, *n* = 593; 1.4%, *n* = 1). Genes with the most relatives per family tested were *APOB* (3.1 relatives/proband) and *PCSK9* (2.5 relatives/proband) in the diagnostic cohort and in *MSH2* (4.5 relatives/proband) and *APOB* (3.7 relatives/proband) in the proactive cohort (Figure 1C).

### HCS Panels: Proband Results and Cascade Testing Outcomes

**FIGURE 2:**
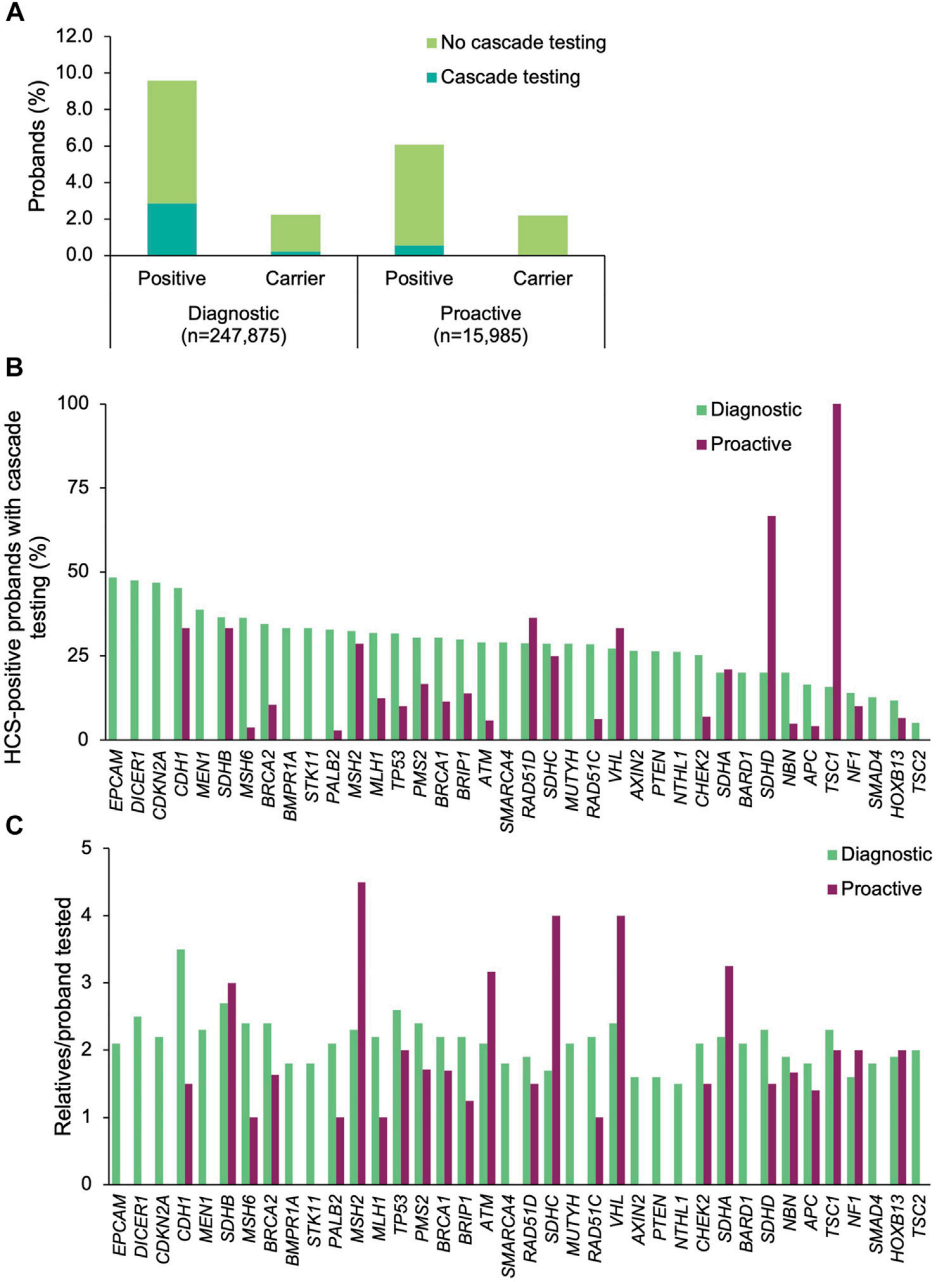
Yield of medically actionable (positive) and clinically significant (carrier) findings in probands who underwent diagnostic testing or proactive screening for HCS genes and rates of cascade testing. **(A)** Proportion of diagnostic and proactive probands with a positive result in an HCS gene, stratified by whether cascade testing was pursued. The denominator was the total number of probands who underwent diagnostic testing (*n* = 247,875) or proactive screening (*n* = 15,985) for HCS genes. **(B)** Proportion of probands with a medically actionable result in each HCS gene common to both diagnostic and proactive panels of interest who had at least one relative undergo cascade testing. The denominator was the number of probands with a positive result in each HCS gene for each cohort. Probands with a positive finding in more than one HCS gene were included in calculations for each gene. Thirty-eight of the 45 shared HCS genes are shown. Data for the remaining seven genes can be found in Supplementary Table S3. **(C)** Mean number of relatives who pursued cascade testing per proband with a positive result in an HCS gene. If cascade testing was pursued for positive findings in more than one gene detected in the proband, the relatives and probands were included in the calculations for each gene. Data are shown for 38 genes; data for the remaining seven genes can be found in Supplementary Table S3. HCS, hereditary cancer syndrome.

Among the 45 shared HCS genes, a positive result was returned to 23,272 (9.4%) of the diagnostic probands and 970 (6.1%) of the proactive probands (Figure 2A). The most common positive findings among diagnostic probands were in *CHEK2* (18.6% of positive findings), *BRCA2* (15.3%), *BRCA1* (11.7%), *ATM* (9.7%), and *APC* (7.3%) (Figure 2B). The most common positive findings among proactive probands were in *CHEK2* (24.0%), *APC* (12.6%), *BRCA2* (10.8%), *ATM* (10.6%), and *BRCA1* (9.1%). The frequencies of positive findings across all HCS genes are listed in Supplementary Table S3.

Cascade testing was pursued significantly more often when a positive finding in an HCS gene was returned for diagnostic probands than when it was returned for proactive probands (diagnostic *n* = 6,611, 28.4%; proactive *n* = 89, 9.2%; *p* = 1.01×10^–43^) (Figure 2A). In general, diagnostic probands were more likely to have at least one relative pursue cascade testing across all HCS genes compared to proactive probands (Figure 2B, Supplementary Table S3). However, cascade testing rates were similar for *MSH2*, *SDHA*, *RAD51D*, *NF1*, *CDH1*, *SDHB*, *SDHC*, and *VHL*. A higher proportion of proactive probands with a medically actionable finding in *SDHD* and *TSC1* had at least one relative undergo cascade testing compared to diagnostic probands, but this difference is likely due to the absolute number of probands in each group that had a medically actionable finding in those genes.

A total of 14,590 relatives of diagnostic probands (2.0 relatives/proband) and 168 relatives of proactive probands (1.9 relatives/proband) were tested. Multigene panel testing was ordered for a minority of relatives (diagnostic *n* = 3,731, 25.6%; proactive *n* = 29, 17.3%), with the remainder having testing limited to genes with clinically significant and/or medically actionable findings in the proband. Most were first-degree relatives (diagnostic 77.2%, *n* = 11,261; proactive 78.0%, *n* = 131), with the remaining being second-degree (10.1%, *n* = 1469; 8.3%, *n* = 14), third-degree (4.7%, *n* = 682; 10.1%, *n* = 17), or more distant relatives (8.1%, *n* = 1,178; 3.0%, *n* = 5). The number of relatives per proband that underwent cascade testing was highest for *CDH1* (3.5 relatives/proband), *SDHB* (2.7 relatives/proband), and *TP53* (2.6 relatives/proband) in the diagnostic cohort and for *MSH2* (4.5 relatives/proband), *SDHC* (4.0 relatives/proband), and *VHL* (4 relatives/proband) in the proactive cohort (Figure 2C).

Relatives in both cohorts were mostly female (diagnostic 68.8%, proactive 68.5%) and self-reported White (diagnostic 73.3%, proactive 62.5%), with a similar mean age at testing (diagnostic 46.4 ± 17.7 years, proactive 44.6 ± 20.6 years) (Table 2). A total of 6,422 (44.0%) and 69 (41.1%) of the relatives of diagnostic and proactive probands, respectively, had at least one clinically significant finding that was consistent with the positive finding in the proband. Additional findings were found in 282 relatives of diagnostic probands, 262 of whom had multigene panel testing. In total, 205 relatives had a finding in another gene on the Common Hereditary Cancers Panel, 45 had a different clinically significant finding in the same gene as the proband’s clinically significant finding, and 32 had a clinically significant finding in a gene that was not analyzed in the proband. One (3.4%) relative of a proactive proband who pursued testing as a result of a positive finding in a shared HCS gene had a positive finding in another gene.

**TABLE 2:** Demographic information of relatives.

	HCS		FH	
	Diagnostic relatives (N = 14,590)	Proactive relatives (N = 168)	Diagnostic relatives (N = 873)	Proactive relatives (N = 13)
Sex, n (%)^a^				
Female	10,039 (68.8)	115 (68.5)	468 (53.6)	7 (53.8)
Male	4,550 (38.2)	53 (31.5)	405 (46.4)	6 (42.2)
Age, years				
Mean (SD)	46.4 (17.7)	44.6 (20.6)	27.6 (19.7)	25.9 (20.5)
Median (Q1, Q3)	6 (33, 60)	42.5 (27, 63)	22 (11, 43)	15 (9, 37)
Self-reported ancestry, n (%)				
Ashkenazi Jewish	367 (2.5)	13 (7.7)	4 (0.5)	0
Asian	336 (2.3)	9 (5.4)	39 (4.5)	10 (76.9)
Black	361 (2.5)	0	18 (2.1)	0
French-Canadian	25 (0.2)	0	1 (0.1)	0
Hispanic	920 (6.3)	14 (8.3)	44 (5.0)	0
Mediterranean	30 (0.2)	3 (1.8)	1 (0.1)	0
Native American	26 (0.2)	0	0	0
Pacific Islander	3 (0.02)	0	0	0
Sephardic Jewish	54 (0.4)	1 (0.6)	0	0
White	10,700 (73.3)	105 (62.5)	642 (73.5)	1 (7.7)
Multiple ancestries	1,061 (7.3)	14 (8.3)	35 (4.0)	2 (15.4)
Other	177 (1.2)	3 (1.8)	28 (3.2)	0
Unknown	530 (3.6)	6 (3.6)	61 (7.0)	0
Relationship to proband				
FDR	11,261 (77.2)	131 (78.0)	702 (80.4)	11 (84.6)
SDR	1469 (10.1)	15 (8.9)	92 (10.5)	2 (15.4)
TDR	682 (4.7)	17 (10.1)	47 (5.4)	0
More distant	1178 (8.1)	5 (3.0)	32 (3.7)	0

FDR, first-degree relative; FH, familial hypercholesterolemia; HCS, hereditary cancer syndrome; Q, quartile; SD, standard deviation; SDR, second-degree relative; TDR, third-degree relative.

a Sex of one diagnostic relative was unknown among diagnostic probands undergoing HCS testing.

**FIGURE 3:**
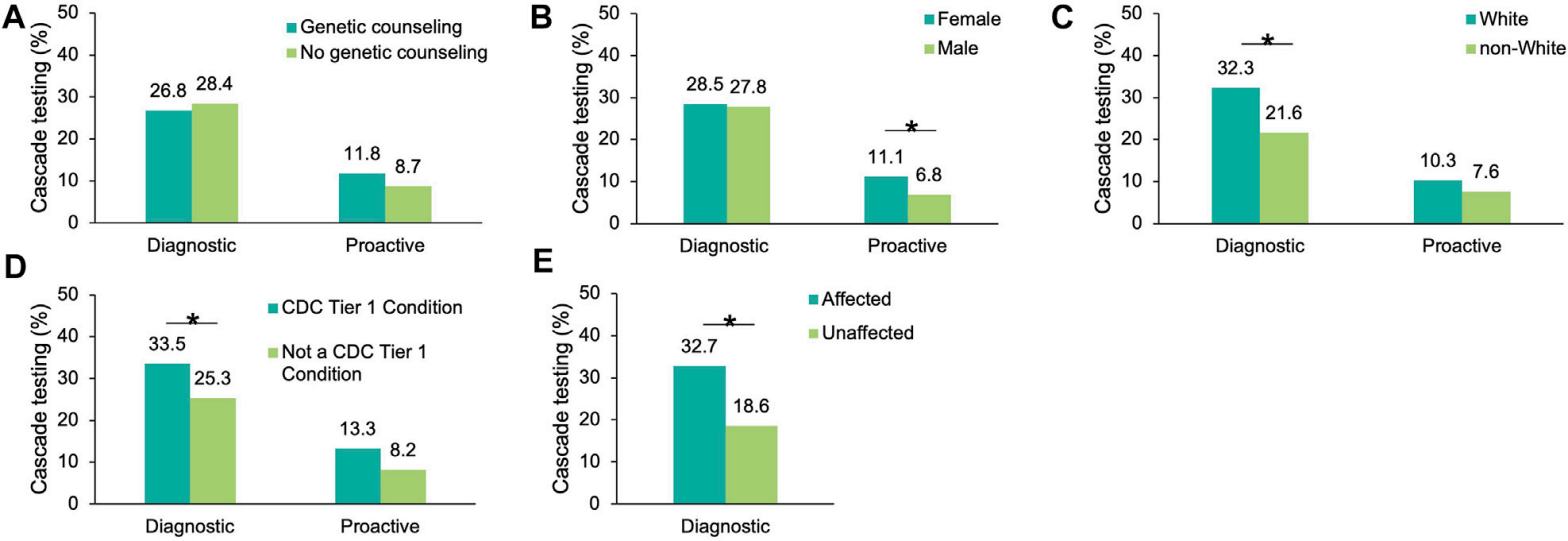
Factors influencing cascade testing among probands with a positive result in an HCS gene. Cascade testing rates were calculated for probands with a positive finding in an HCS gene and stratified by proband characteristic: **(A)** Whether the proband had genetic counseling through Invitae, **(B)** Sex **(C)** Self-reported ancestry, **(D)** Whether the positive finding in the proband was in a gene associated with a CDC Tier 1 condition, and **(E)** Whether diagnostic probands reported a family or personal history of cancer (this information was not available for proactive probands). HCS, hereditary cancer syndrome.

To understand which factors may increase the likelihood of cascade testing, the differences in cascade testing uptake rates among probands with a positive result were compared based on demographics (Supplementary Table S4), whether genetic counseling services through Invitae were utilized, whether the finding was in a gene associated with a CDC Tier 1 condition, and whether a personal history of cancer was reported (diagnostic probands only). No differences in genetic counseling utilization were observed for either cohort (Figure 3A). Cascade testing was more commonly pursued among proactive probands who were female (11.1 *vs*. 6.8%, *p* = 0.019), (Figure 3B). It was also pursued more frequently among diagnostic probands who were White (32.3 *vs*. 21.6%, *p* = 2.49×10^–69^) (Figure 3C), had a gene finding associated with a CDC Tier 1 condition (33.5 *vs*. 25.3%, *p* = 5.91×10^–41^) (Figure 3D), or had a personal history of cancer (32.7 *vs*. 18.6%, *p* = 4.52×10^–108^) (Figure 3E).

A small proportion of diagnostic (*n* = 5,559, 2.2%) and proactive (*n* = 350, 2.2%) probands had carrier results returned (in the genes included in this analysis) (Figure 2A). At least one relative of 9.4% (*n* = 524) and 1.7% (*n* = 6) of the diagnostic and proactive probands, respectively, had cascade testing performed as a result of carrier findings.

### FH Panels: Proband Results and Cascade Testing Outcomes

**FIGURE 4:**
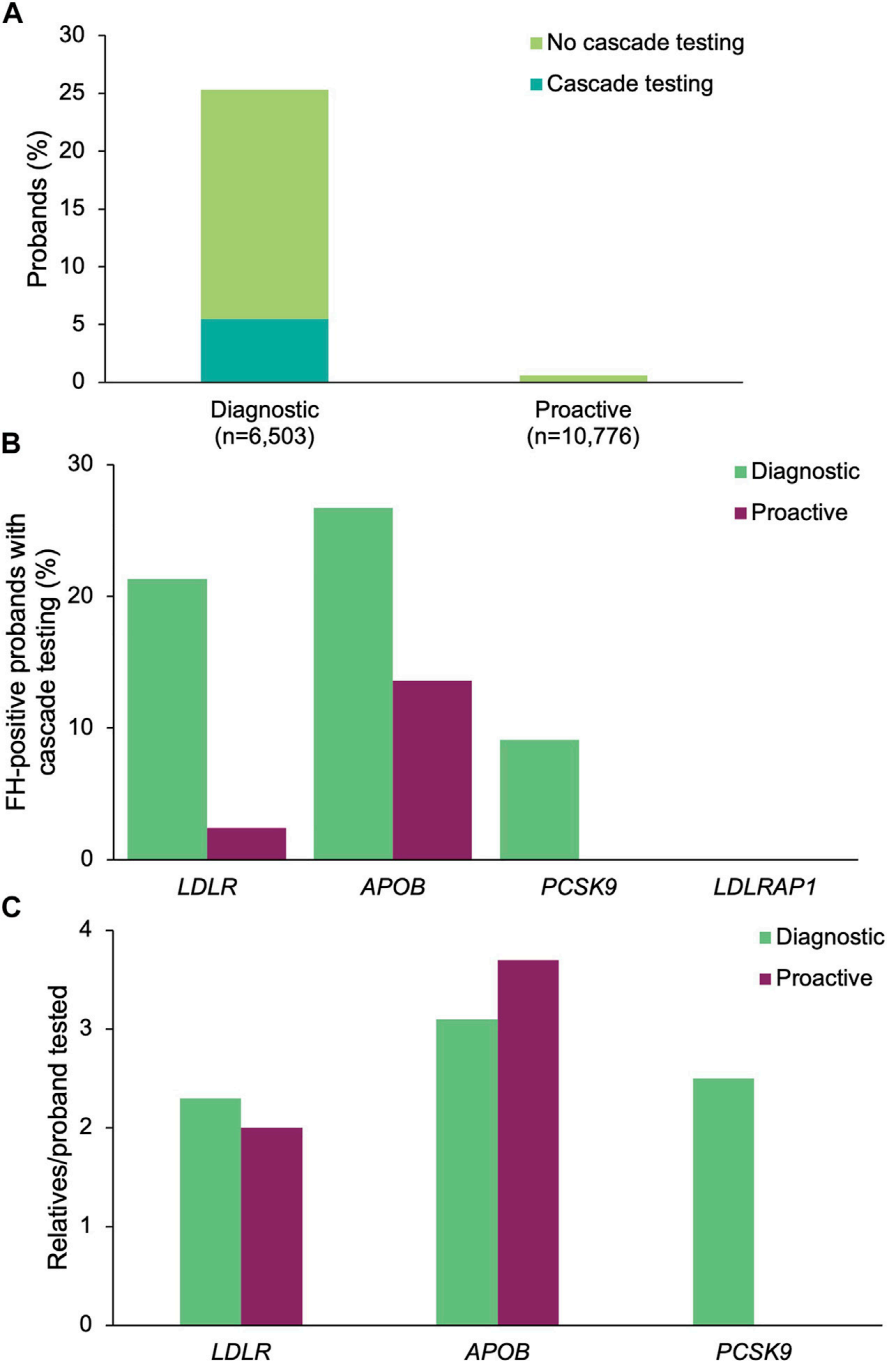
Yield of medically actionable (positive) findings in probands who underwent diagnostic testing or proactive screening for FH genes and rates of cascade testing. **(A)** Proportion of diagnostic and proactive probands with a positive result in an FH gene, stratified by whether cascade testing was pursued. The denominator was the total number of probands who underwent diagnostic testing (*n* = 6,503) or proactive screening (*n* = 10,776) for FH genes. **(B)** Proportion of probands with a positive result in each FH gene common to both diagnostic and proactive panels of interest who had at least one relative pursue cascade testing. The denominator was the number of probands with a positive result in each FH gene for each cohort. Probands with a positive finding in more than one FH gene were included in calculations for each gene. **(C)** Mean number of relatives who pursued cascade testing per proband with a positive result in an FH gene. If cascade testing was pursued for positive findings in more than one gene detected in the proband, the relatives and probands were included in the calculations for each gene. FH, familial hypercholesterolemia.

A positive result in at least one of the four FH genes was returned to 1,647 (25.3%) of the diagnostic probands and 67 (0.62%) of the proactive probands (Figure 4A). The most common positive findings among diagnostic probands were in *LDLR* (86.4%), *APOB* (12.3%), *PCSK9* (1.3%), and *LDLRAP1* (0.3%). The most common positive findings among proactive probands were in *LDLR* (62.7%), *APOB* (32.8%), *PCSK9* (4.5%). No proactive probands had a positive finding in *LDLRAP1*, though four probands were carriers (see below).

A positive finding in an FH gene in 360 (21.9%) of the diagnostic probands and 4 (6.0%) of the proactive probands led to cascade testing in at least one relative (*p* = 0.00183) (Figure 4A). Cascade testing was pursued in relatives of diagnostic probands with positive findings in *LDLR* (*n* = 304, 21.3%), *APOB* (*n* = 54, 26.7%), and *PCSK9* (*n* = 2, 9.1%) (Figure 4B). Proactive probands with positive findings in *APOB* (*n* = 3, 13.6%) and *LDLR* (*n* = 1, 2.4%) led to cascade testing. A total of 873 relatives of diagnostic probands (2.4 relatives/proband) and 13 relatives of proactive probands (3.3 relatives/proband) were tested, of whom 37 (4.2%) diagnostic relatives and 2 (15.4%) proactive relatives had multigene panels ordered. The remainder had testing limited to genes with clinically significant and/or medically actionable findings in the proband. Relatives who underwent cascade testing were mostly first-degree relatives (diagnostic 80.4%, *n* = 702; proactive 84.6%, *n* = 11), with the remaining reported to be second-degree (10.5%, *n* = 92; 15.4%, *n* = 2), third-degree (5.4%, *n* = 47; *n* = 0), or more distant related (3.7%, *n* = 32; *n* = 0). The number of relatives per proband who underwent cascade testing was highest for *APOB* (3.1 relatives/proband), *PCSK9* (2.5 relatives/proband), and *LDLR* (2.3 relatives/proband) in the diagnostic cohort and for *APOB* (3.7 relatives/proband) and *LDLR* (2.0 relatives/proband) in the proactive cohort (Figure 4C). Demographic characteristics of relatives in both cohorts were similar (Table 2). A total of 496 (56.8%) and 4 (30.8%) relatives of diagnostic or proactive probands, respectively, had a positive finding, all of which were consistent with the positive finding in the proband. No relatives of diagnostic or proactive probands who pursued testing as a result of a medically actionable finding in a shared FH gene had a positive finding in another gene. Two relatives of diagnostic probands were carriers for *ABCG8* and one relative of a proactive proband who had a positive finding in *APOB* was also identified as a carrier for *RYR1*. Two (5.4%) diagnostic relatives and zero proactive relatives who had multigene panel testing had a clinically significant finding returned outside of the proband’s diagnostic testing or proactive screening results.

**FIGURE 5:**
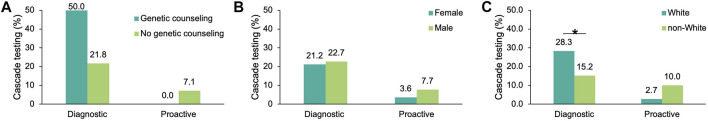
Factors influencing cascade testing rates among probands with a positive result in an FH gene. Cascade testing rates were calculated for probands with a positive finding in an FH gene and stratified by characteristic: **(A)** Whether the proband had genetic counseling through Invitae, **(B)** Sex, and **(C)** Self-reported ancestry. FH, familial hypercholesterolemia.

To understand which factors may increase the likelihood of cascade testing, the differences in cascade testing uptake rates among probands with a positive result were compared based on demographics (Supplementary Table S5) and whether post-test genetic counseling services through Invitae were utilized. Cascade testing was more commonly pursued in proactive probands who had genetic counseling (11.8 *vs*. 8.7%, Figure 5A), were male (7.7 *vs*. 3.6%, Figure 5B), or non-White (10.1 *vs.* 8.0%, Figure 5C). Results in the proactive cohort should be interpreted with caution as the sample size of proactive probands with cascade testing was small (*n* = 4). Cascade testing was pursued more frequently among diagnostic probands who were younger at time of testing (29.4 ± 19.8 years *vs*. 38.6 ± 22.7 years, *p* = 1.28 × 10^–13^) or self-reported White (28.3 *vs*. 15.2%, *p* = 1.36 × 10^–10^) (Figure 5C).

A small proportion of diagnostic (*n* = 3, 0.05%) and proactive (*n* = 4, 0.04%) probands had carrier results returned. No relatives pursued cascade testing as a result of these carrier findings in either cohort.

### Findings in Additional Genes Unique to the Diagnostic and Proactive Panels

In addition to the 49 genes that were available on both diagnostic and proactive gene panels of interest, an additional two genes (*RAD50* and *CTNNA1*) were available only on diagnostic panels. A small number (626, 0.3%) of diagnostic probands who had testing via the Invitae Common Hereditary Cancers Panel had a positive result in *RAD50* (no probands had a positive result in *CTNNA1*), 108 (17.3%) of whom had 205 relatives (1.9 relatives/proband) pursue cascade testing.

An additional 98 genes were available only on the proactive panels, including genes associated with HCS (*n* = 16), other non-FH cardiology conditions (*n* = 73), or other conditions (*n* = 10) (Supplementary Table S1). The proportion of positive results in one of these genes ranged from 0.6% in HCS genes to 9.2% in cardiology genes (Supplementary Figure S1). Cascade testing was most commonly pursued for positive findings in an HCS gene (HCS 10.8%, cardiology 2.4%, other clinical areas 1.6%).

## Discussion

In addition to the potential utility of genetic testing results to inform an individual’s clinical care and outcomes, a positive result has implications for that individual’s family. Studies assessing the uptake of cascade testing among relatives in a diagnostic setting have consistently demonstrated that rates are generally low, though they vary based on clinical area and focus only on just a few genes (Fehniger et al., 2013; Menko et al., 2019; Ajufo et al., 2021; Lee et al., 2021). As population-based and proactive screening methods begin to become more widespread, it is critical to understand how these testing approaches may impact at-risk relatives. Currently, the utilization of cascade testing in a non-indication-based, proactive setting is less well understood and uptake rates have not yet been reported. This study compared findings between two cohorts that differed in how NGS was pursued: indication-based diagnostic testing versus non-indication-based proactive screening. The findings reported allow not only for insights into differences between diagnostic and proactive results, but also more generally to ordering patterns for diagnostic testing or proactive screening for HCS and FH. Interestingly, we also gain tangential and preliminary insights into the potential benefits of multigene panel testing in at-risk relatives. This study demonstrates that there is an even larger gap in the uptake of cascade testing in a proactive versus diagnostic setting and highlights the need for further research to understand both the reasons for underutilization of cascade testing and the approaches that could lead to increased uptake rates.

In this study, we find that cascade testing rates were significantly higher among diagnostic probands compared to proactive probands across all comparisons, including testing or screening for any CDC Tier 1 condition, for HCS, and for FH. The findings from this study are the first to begin to investigate which factors may be associated with cascade testing utilization in a proactive setting. Proband characteristics shown to be associated with cascade testing in a diagnostic setting were consistent with our cohort, including self-reported ancestry, sex, and a personal history of disease (Dugan et al., 2003; Hamilton et al., 2005; Gaff et al., 2007; Sharaf et al., 2013; Roberts et al., 2018; Caswell-Jin et al., 2019; Menko et al., 2019; Braley et al., 2021). The only factor that resulted in a significant difference in cascade testing rates in the proactive cohort was sex, with rates higher among female probands. These preliminary findings demonstrate that there may be different factors that influence the utilization of cascade testing depending on the method of testing in the index case. Further prospective studies exploring a wider variety of proband and relative characteristics in relation to cascade testing rates will be critical to developing tools for encouraging and facilitating cascade testing that are tailored to various testing methods (i.e., diagnostic versus proactive).

Two large hurdles must be overcome in order for cascade testing to be pursued; first, the proband must share results with at-risk relatives and second, the relative must make the choice to seek genetic testing. It has been established that results sharing is poor regardless of whether diagnostic testing or proactive screening is ordered (Dugan et al., 2003; Hamilton et al., 2005; Gaff et al., 2007; Elrick et al., 2017; Wurtmann et al., 2018). Reasons for a lack of cascade testing utilization is limited to a diagnostic setting, with no research yet focusing on potential barriers for proband testing in a proactive setting. However, it is likely some of the reasons are similar for both approaches. Diagnostic probands have cited a perceived lack of clinician support and familial relationships as barriers (Dugan et al., 2003; Chivers Seymour et al., 2010; Muir et al., 2012; Hardcastle et al., 2015; Pollard et al., 2020; Srinivasan et al., 2020). Among the limited pool of at-risk relatives who do have results shared with them, only a small proportion end up seeking cascade testing. Previous research has shown that relatives of diagnostic probands do not seek testing because of several perceived hurdles, including cost, the need to make an appointment with a clinician, and concerns about insurance or employment discrimination, even with current legislation barring such discrimination (EEOC, 2008; Hendricks-Sturrup et al., 2019; Srinivasan et al., 2020).

Approaches to encouraging and facilitating cascade testing have been largely limited to a diagnostic setting. However, ongoing studies, such as the IMPACT-FH (Identification Methods, Patient Activation, and Cascade Testing for FH) study (Campbell-Salome et al., 2021), are exploring strategies that increase the uptake of cascade testing in population-based screening programs. However, learnings from the diagnostic setting may provide some insights, including the availability of clinician-drafted letters (Newson and Humphries, 2005; Suthers et al., 2006; Hadfield et al., 2009; Dilzell et al., 2014; Petersen et al., 2019; Kurian and Katz, 2020; Neuner et al., 2020), access to support from foundations focused on a single condition or clinical area (Bell et al., 2015; Wald et al., 2016; McGowan et al., 2021), and access to educational materials that are easily shared outside of a clinical setting (Kardashian et al., 2012; Petersen et al., 2019; Bowen et al., 2020; Jujjavarapu et al., 2021; Nazareth et al., 2021; Nitecki et al., 2021; Snir et al., 2021). When considering approaches to encouraging at-risk relatives to ultimately seek cascade testing, programs have been designed to offer cascade testing at reduced rates or at no-charge for relatives (Aktan-Collan et al., 2007; Caswell-Jin et al., 2019; Courtney et al., 2019; Invitae, 2021). Preliminary findings in a recent study demonstrated that chatbots are an effective means to facilitating cascade testing (data in press Schmidlen et al., 2022). Regardless of the approach, it is critical that methods used to encourage both results sharing and subsequent cascade testing are accessible to diverse populations (Milo Rasouly et al., 2021).

The sum of these observations demonstrates that there is not likely a one size fits all approach to encouraging cascade testing, and that having several avenues available for both facilitating results sharing and streamlining testing processes will maximize the success of cascade testing initiatives. Especially for probands who are identified in non-indication-based settings, additional efforts to educate probands, as well as tools to help them share information with relatives, will be essential as genetic screening in healthy individuals becomes more widespread. For example, novel approaches utilizing chatbots may not only improve communication with probands but also facilitate results sharing and subsequently help connect relatives to a clinician for cascade testing. This is especially important as genetic counseling may not be sought prior to or after screening in the proband.

In addition to insights into differences between diagnostic and proactive cohorts, ordering behaviors among probands seeking testing or screening for HCS and FH were very different. Strikingly, the absolute number of probands who were tested for HCS and FH panels was very different for both the diagnostic and proactive cohorts. It is possible that this is due to the increased awareness of and testing for hereditary breast and ovarian cancer and Lynch syndrome compared to that of FH. While we observe higher cascade testing uptake for HCS, the number of relatives tested per proband is higher for FH compared to HCS (∼3 relatives/proband vs. 2 relatives/proband). We suspect that this is because genetics specialists are providing care to probands being tested for FH in collaboration with the treating clinician (Ingles et al., 2020; Musunuru et al., 2020), while oncologists may have an increased experience and comfort with ordering genetic testing themselves (Hamilton et al., 2021). So while more probands could be referred for HCS testing by a non-genetics specialist, probands tested for FH may more likely be receiving counseling from genetic counselors and as a result, may have higher numbers of relatives tested once results are shared.

Finally, though limited to a minority of relatives, anywhere from ∼5 to ∼25% of relatives have additional genes (often multigene panel testing) ordered. Among relatives who had probands undergo diagnostic testing or proactive screening for HCS, 7.0% of relatives of diagnostic probands and 3.4% of relatives of proactive probands had a clinically significant finding outside of the proband’s findings. This finding demonstrates that panel testing does in fact identify additional risks that would have otherwise been missed had gene-specific testing been ordered. Reasons for missing these clinically significant findings could be a result of, among others, the proband not being tested for that gene or that the relative has a family history associated with another relative unrelated to the proband. Relatives who received a negative result following targeted testing based on proband results could have a false sense of reassurance without understanding that they could have medically actionable variants in other genes. This may be the case even though clinicians take into account an individual’s full family history and genetic counseling based on a negative result centers around residual risk. While there may be higher costs related to testing for additional genes, these results underscore the possible benefits of considering broader testing for relatives seeking cascade testing.

Similar to other retrospective cohort studies, this study was limited in the data available for analysis. Our analysis compared two cohorts based on how probands were referred: indication-based diagnostic testing or non-indication-based proactive screening. As a commercial testing laboratory, orders are received from clinicians requesting diagnostic testing as well as individuals seeking proactive screening. The majority of individuals in these cohorts indicated a self-reported White ancestry, which may have biased the results. The socioeconomic factors demonstrated to impact the utilization of genetic testing were not controlled for in this study (Gómez-Trillos et al., 2020; McKinney et al., 2020; Giri et al., 2021). While not a focus in this study, Invitae has sponsored testing programs that eliminate potential financial barriers to diagnostic genetic testing for a number of clinical indications, in addition to research initiatives to help facilitate population screening and cascade testing across more diverse groups (staff reporter, 2021). Novel approaches to improving genetics literacy and awareness across diverse populations have proven to be successful (Milo Rasouly et al., 2021). Among diagnostic probands, the specific reason for testing could not be determined because the test requisition form did not require disclosure of whether the individual for whom testing was ordered had a personal or family history of an HCS or FH. Thus, whether a proband had a personal or family history was unknown for many individuals and was not uniform when shared. However, for a number of hereditary cancer conditions and familial cardiac conditions, current guidelines recommend that family history alone, when meeting certain requirements, is a standalone indication for diagnostic genetic testing in an otherwise unaffected individual (e.g., family history of breast cancer in multiple first degree relatives) (Daly et al., 2021). For these logistic and clinical reasons, we could only assume that diagnostic testing was warranted based on the ordering clinician’s evaluation of the individual. Another limitation is that, as the testing laboratory, the total number of relatives that were offered cascade testing could not be determined. As such, the cascade testing uptake rate is based purely on those individuals tested through Invitae with reported relationships disclosed at the time of test requisition. The number of probands with clinically significant results who shared their results with relatives and the number of relatives who ultimately sought testing could not be determined. However, as reported from other studies, it is clear that results sharing and subsequent testing rates are generally low. Further, it is unknown how many probands sought genetic counseling outside of Invitae. It is expected that most diagnostic probands, but far fewer proactive probands, had received counseling through a clinician or an adjacent clinical service (such as a medical geneticist or genetic counselor). However, this information was not well documented, so assessing the rate of cascade testing based on genetic counseling through Invitae may be an underestimate. Although limited, this study helps to establish preliminary findings that can help to guide future prospective studies.

The results of this study have demonstrated that cascade testing uptake is significantly lower among probands who seek testing in a non-indication-based, proactive setting than among those who are referred for indication-based testing. The barriers and facilitators of cascade testing seem to be similar between the two cohorts, suggesting that approaches that promote family testing in a diagnostic setting could be similarly applied to proactive settings. However, the tools and methods may need to be tailored to these different settings in order to increase cascade testing rates. Such an investigation is underway in individuals undergoing testing for FH as part of a population-based genomic research study (Campbell-Salome et al., 2021). The findings from the present study establish a baseline for future prospective studies designed to understand the reasons for results sharing (or not) among probands and the subsequent influences that encourage relatives to engage with cascade testing.

## Data Availability

The original contributions presented in the study are included in the article/Supplementary Material, further inquiries can be directed to the corresponding author.
