# Supplementary material for: The Impact of Proband Indication for Genetic Testing on the Uptake of Cascade Testing Among Relatives
Source: Front Genet. 2022 Jun 16;13:867226. doi: 10.3389/fgene.2022.867226 (PMC9243226; doi:10.3389/fgene.2022.867226)
Supplement: Supplementary file 1 [file Table2.DOCX]

**Supplementary Material for: The Impact of Proband Indication for Genetic Testing on the Uptake of Cascade Testing Among Relatives**

**Table S1.** Genes included in the analysis (see Excel sheet)

**Table S2.** Demographic information for all probands in the diagnostic and proactive cohorts

**Table S3.** Summary of positive findings in HCS genes among probands and resulting cascade testing in relatives (see Excel sheet)

**Table S4.** Characteristics of probands who underwent NGS for HCS with a positive finding, stratified by cascade testing

**Table S5.** Characteristics of probands who underwent NGS for FH with a positive finding, stratified by cascade testing

**Table S1. Genes included in the analysis**

See Excel sheet

**Table S2. Demographic information for all probands in the diagnostic and proactive cohorts**

|  | Diagnostic probands  (N=254,281) | Proactive probands  (N=16,434) |
| --- | --- | --- |
| Sex, n (%)^a^     Female     Male | 220,569 (86.7)  33,710 (13.3) | 9,517 (57.9)  6,917 (42.1) |
| Age, years     Mean (SD)     Median (Q1, Q3) | 55.2 (14.7)  56 (45, 67) | 48.3 (13.2)  48 (38,58) |
| Self-reported ancestry, n (%)     Ashkenazi Jewish     Asian     Black     French-Canadian     Hispanic     Mediterranean     Native American     Pacific Islander     Sephardic Jewish     White     Multiple ancestries     Other     Unknown | 7,720 (3.0)  8,325 (3.3)  17,230 (6.8)  334 (0.1)  17,958 (7.1)  707 (0.3)  549 (0.2)  366 (0.1)  276 (0.1)  164,400 (64.5)  21,084 (8.3)  4,050 (1.6)  11,582 (4.6) | 621 (3.8)  1,096 (6.7)  239 (1.5)  33 (0.2)  447 (2.7)  152 (0.9)  10 (0.1)  15 (0.1)  110 (0.7)  9,976 (60.7)  1,566 (9.5)  740 (4.5)  1,429 (8.7) |
| NGS panel type, n (%)     Cancer     Cardiology     Both | 247,778 (97.4)  6,406 (2.5)  97 (0.04) | 5,659 (34.4)  450 (2.7)  10,326 (62.8) |

HCS, hereditary cancer syndrome; Q, quartile; SD, standard deviation.

^a^Sex was unknown for two diagnostic probands undergoing HCS testing.

**Table S3. Summary of positive findings in HCS genes among probands and resulting cascade testing in relatives**

See Excel sheet

**Table S4. Characteristics of probands who underwent NGS for HCS with a positive finding, stratified by cascade testing**

|  | Diagnostic | | Proactive | |
| --- | --- | --- | --- | --- |
|  | Cascade testing  (n=6,611) | No cascade testing  (n=16,661) | Cascade testing  (n=89) | No cascade testing  (n=881) |
| Sex, n (%)     Female     Male | 5,495 (83.1)  1,116 (16.9) | 13,761 (82.6)  2,900 (17.4) | 59 (66.3)  30 (33.7) | 470 (53.3)  411 (46.7) |
| Age, years     Mean (SD)     Median (Q1, Q3) | 56.0 (14.5)  57 (45, 66) | 54.5 (15.2)  55 (43, 66) | 49.4 (14.5)  46 (38, 59) | 47.5 (13.4)  46 (37, 57) |
| Self-reported ancestry, n (%)     Ashkenazi Jewish     Asian     Black     French-Canadian     Hispanic     Mediterranean     Native American     Pacific Islander     Sephardic Jewish     White     Multiple ancestries     Other     Unknown | 214 (3.2)  161 (2.4)  181 (2.7)  8 (0.1)  349 (5.3)  16 (0.2)  11 (0.2)  2 (0.03)  5 (0.1)  4,778 (72.3)  551 (8.3)  73 (1.1)  262 (4.0) | 933 (5.6)  576 (3.5)  1,010 (6.1)  12 (0.1)  1,262 (7.6)  50 (0.3)  41 (0.3)  19 (0.1)  15 (0.1)  9,989 (60.0)  1,523 (9.1)  287 (1.7)  944 (5.7) | 5 (5.6)  3 (3.4)  0  0  7 (7.9)  0  0  0  1 (1.1)  58 (65.2)  9 (10.1)  0  6 (6.7) | 80 (9.1)  36 (4.1)  10 (1.1)  0  22 (2.5)  10 (1.1)  0  0  9 (1.0)  503 (57.1)  99 (11.2)  46 (5.2)  66 (7.5) |

HCS, hereditary cancer syndrome; NGS, next generation sequencing; Q, quartile; SD, standard deviation.

**Table S5. Characteristics of probands who underwent NGS for FH with a positive finding, stratified by cascade testing**

|  | Diagnostic | | Proactive | |
| --- | --- | --- | --- | --- |
|  | Cascade testing  (n=360) | No cascade testing  (n=1,287) | Cascade testing  (n=4) | No cascade testing  (n=63) |
| Sex, n (%)     Female     Male | 193 (53.6)  167 (46.1) | 718 (55.8)  569 (44.2) | 1 (25.0)  3 (75.0) | 27 (42.9)  36 (57.1) |
| Age, years     Mean (SD)     Median (Q1, Q3) | 29.4 (19.8)  36 (18, 55) | 38.6 (22.7)  24 (11, 44) | 50.5 (9.3)  50 (45.3, 55.3) | 47.2 (10.9)  46 (38.5, 55) |
| Self-reported ancestry, n (%)     Ashkenazi Jewish     Asian     Black     French-Canadian     Hispanic     Mediterranean     Native American     Pacific Islander     Sephardic Jewish     White     Multiple ancestries     Other     Unknown | 3 (0.8)  18 (5.0)  11 (3.0)  0  29 (8.0)  0  0  0  0  237 (65.8)  21 (5.8)  10 (2.8)  31 (8.6) | 9 (0.7)  84 (6.5)  72 (5.6)  6 (0.5)  164 (12.8)  13 (1.0)  4 (0.3)  2 (0.2)  2 (0.2)  601 (46.7)  86 (6.7)  31 (2.4)  213 (16.6) | 0  1 (25.0)  0  0  0  0  0  0  0  1 (25.0)  0  1 (25.0)  1 (25.0) | 1 (1.6)  6 (9.5)  1 (1.6)  0  3 (4.8)  0  0  0  0  36 (57.1)  3 (4.8)  2 (3.2)  11 (17.5) |

FH, familial hypercholesterolemia; NGS, next generation sequencing; Q, quartile; SD, standard deviation.

**
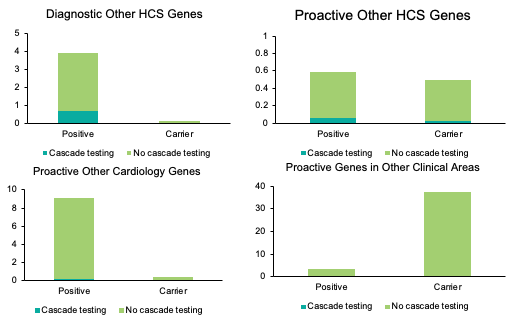
**

**Supplementary Figure S1. Proportion of probands with a finding in a gene unique to the diagnostic or proactive multigene panels of interest.**
